# Prevalence of anti‐severe acute respiratory syndrome coronavirus 2 antibodies in cats in Germany and other European countries in the early phase of the coronavirus disease‐19 pandemic

**DOI:** 10.1111/zph.12932

**Published:** 2022-03-02

**Authors:** Julia Maria Adler, Corinna Weber, Kerstin Wernike, Anna Michelitsch, Karin Friedrich, Jakob Trimpert, Martin Beer, Barbara Kohn, Klaus Osterrieder, Elisabeth Müller

**Affiliations:** ^1^ Institut für Virologie Freie Universität Berlin Berlin Germany; ^2^ Laboklin GmbH & Co.KG Bad Kissingen Germany; ^3^ Institute of Diagnostic Virology Friedrich‐Loeffler‐Institut Greifswald–Insel Riems Germany; ^4^ Klinik für kleine Haustiere Freie Universität Berlin Berlin Germany; ^5^ Department of Infectious Diseases and Public Health Jockey Club College of Veterinary Medicine and Life Sciences City University of Hong Kong Kowloon Hong Kong; ^6^ Present address: Department of Infectious Diseases and Respiratory Medicine Charité Universitätsmedizin Berlin Berlin Germany

**Keywords:** cats, COVID‐19, SARS‐CoV‐2, seroprevalence

## Abstract

During the first months of the coronavirus disease (COVID‐19) pandemic caused by severe acute respiratory syndrome coronavirus 2 (SARS‐CoV‐2), cases of human‐to‐cat transmission were reported. Seroconversion was shown in cats infected under experimental and natural conditions. This large‐scale survey of 1,005 serum samples was conducted to investigate anti‐SARS‐CoV‐2 antibody prevalence in domestic cats during the first 7 months of the pandemic in Germany and other European countries. In addition, we compared the sensitivity and specificity of two multispecies SARS‐CoV‐2 antibody enzyme‐linked immunosorbent assays (ELISA). Results were confirmed by using an indirect immunofluorescence test (iIFT) and a surrogate virus neutralization test (sVNT). Sera that were highly positive for feline coronavirus (FCoV) antibodies (*n* = 103) were included to correct for cross‐reactivity of the tests used. Our results showed an overall SARS‐CoV‐2 seropositivity of 1.9% (*n* = 19) in a receptor‐binding domain (RBD)‐based ELISA, additional 0.8% (*n* = 8) were giving inconclusive results. In contrast, a nucleocapsid‐based ELISA revealed 0.5% (*n* = 5) positive and 0.2% (*n* = 2) inconclusive results. While the iIFT and sVNT confirmed 100% of positive and 50%–57.1% of the doubtful results as determined in the RBD ELISA, the nucleocapsid‐based assay showed a high discrepancy and only one of the five positive results could be confirmed. The results indicate significant deficits of the nucleocapsid‐based ELISA with respect to sensitivity and specificity. Due to a significantly higher rate (5.8%) of positive results in the group of highly FCoV antibody‐positive samples, cross‐reactivity of the FCoV‐ELISA with SARS‐CoV‐2 antibodies cannot be excluded. Furthermore, we investigated the impact of direct contact of domestic cats (*n* = 23) to SARS‐CoV‐2 positive owners. Considering one inconclusive result, which got confirmed by iIFT, this exposure did not lead to a significantly higher prevalence (4.4%; *p* = .358) among tested subjects. Overall, we conclude that cats are a negligible entity with respect to virus transmission in Europe.

**Impacts**

- A screening for anti‐severe acute respiratory syndrome coronavirus 2 (SARS‐CoV‐2) antibodies in domestic cats during the early phase of the coronavirus disease‐19 pandemic revealed an overall seropositivity of 1.9%, while 0.8% yielded inconclusive results using an RBD‐based ELISA.
- Reported direct exposure of cats to SARS‐CoV‐2‐positive owners led to higher seroprevalence (4.4%), which was not statistically significant.
- The comparison of two anti‐SARS‐CoV‐2 antibody ELISAs (RBD‐ and nucleocapsid‐based) tested for possible cross‐reactivity to feline coronavirus showed deficits in sensitivity and specificity of the nucleocapsid‐based assay.

## 1 INTRODUCTION

Roughly, 36% of households in Germany are keeping dogs or cats as companion animals (Headey & Grabka, 2007). Within the last decades, the relationship between humans and pets is characterized by more intensive contact (Franklin & White, 2001; Krahn et al., 2015). Therefore, it is reasonable to assume that the latter may be conducive to zoonotic transmission of infectious agents. Thus, One Health concepts targeting pets gain importance as does the identification of pathogens with zoonotic potential (Cantas & Suer, 2014; Kahn et al., 2007).

In December 2019, a novel respiratory disease caused by a pathogen known as severe acute respiratory syndrome coronavirus 2 (SARS‐CoV‐2) has occurred in humans in Wuhan, China (Zhu, Zhang, et al., 2020). Genomic similarities suggest coronaviruses of bats or pangolins as proximal if not direct sources of SARS‐CoV‐2 transmission to humans (Xiao et al., 2020; Zhang, Wu, et al., 2020). While knowledge about intermediate hosts and reservoir animals was lacking at the beginning of the pandemic, susceptibility of companion animals soon became a focus of interest. Experimental infections confirmed moderate to high susceptibility of cats to SARS‐CoV‐2 infection, whereas dogs were reported to be less susceptible (Bosco‐Lauth et al., 2020; Shi et al., 2020). In cats, experimental SARS‐CoV‐2 infections usually remain asymptomatic. In naturally infected felids, mild‐to‐moderate respiratory symptoms, nausea and diarrhoea were described (Hossain et al., 2021; Michelitsch, Wernike, et al., 2021). In addition, prolonged virus shedding was reported in felids when compared with dogs (Bosco‐Lauth et al., 2020; Schulz et al., 2021). Contact experiments confirmed the possibility of cat‐to‐cat transmission and thus the potential spread of SARS‐CoV‐2 within feline populations (Bosco‐Lauth et al., 2020; Gaudreault et al., 2020; Halfmann et al., 2020; Shi et al., 2020). Due to the close contact of cats to their owners, various cases of human‐to‐animal transmissions have been reported (Garigliany et al., 2020; Sailleau et al., 2020; Segalés et al., 2020). Zooanthroponotic infection has only been detected in mink farms so far (Munnink et al., 2021), but such examples suggest the possibility of animal‐to‐human transmission.

Seroconversion in domestic cats was shown under experimental and natural conditions (Shi et al., 2020; Zhang, Zhang, et al., 2020). A study conducted in Wuhan, China, revealed a high prevalence (14.7%) of antibodies against the pandemic virus among a population of cats. A small cohort (*n* = 102) of felids from shelters, a veterinary clinic and coronavirus disease (COVID‐19) positive owners was used for this investigation (Zhang, Zhang, et al., 2020). In contrast, the prevalence in Europe based on larger sample numbers seems to be significantly lower for randomly chosen sera (Michelitsch et al., 2020) and in a study including samples from felids with SARS‐CoV‐2‐positive owners (Patterson et al., 2020). Surveys investigating the influence of direct contact of cats to infected owners suggest a rather low anthropozoonotic transmission rate. Whereas in some studies, higher SARS‐CoV‐2 antibody prevalence in cats from COVID‐19 households was found (Fritz et al., 2021; Patterson et al., 2020), other surveys could not detect any signs of seroconversion in felids with direct contact to owners that had tested positive for SARS‐CoV‐2 (Temmam et al., 2020).

To determine seroprevalence of anti‐SARS‐CoV‐2 antibodies in cats, potential cross‐reactivity with endemic coronaviruses must be considered. Feline coronavirus (FCoV) is widespread within the cat population. Infected cats mostly remain asymptomatic carriers (Klein‐Richers et al., 2020), only up to 12% develop feline infectious peritonitis (FIP) (Addie et al., 2009; Foley et al., 1997). Up to 90% of cats kept in catteries and up to 41% in randomly chosen populations carry FCoV‐specific antibodies depending on the population and test design chosen (Loeffler et al., 1977; Pedersen, 1976). Therefore, cross‐reactivity of the tests used must be excluded in order to gain valid results.

Within the first months of the pandemic various ELISAs targeting the nucleocapsid, S1 (spike protein subdomain 1) or the receptor‐binding domain (RBD) have been developed and validated. It was shown that RBD and S1 targeted assays are less susceptible to cross‐reactivity with endemic coronaviruses in humans (Amanat et al., 2020; Chia et al., 2020). These findings were confirmed for endemic coronaviruses in animals as well (Kim et al., 2020).

The aim of this study was to determine the prevalence of anti‐SARS‐CoV‐2 antibodies in cats in Germany and other European countries in the early stages of the pandemic. In addition, a commercially available nucleocapsid‐based multispecies ELISA was tested to compare sensitivity and specificity to a previously published RBD‐based ELISA. Sera known as highly positive for FCoV antibodies were included to account for cross‐reactivity. A cohort (*n* = 23) of cats with owners that tested positive for SARS‐CoV‐2 by RT‐PCR and/or ELISA was targeted to further evaluate influence of direct contact to SARS‐CoV‐2 infected humans.

## 2 MATERIALS AND METHODS

### 2.1 Sampling

**FIGURE 1:**
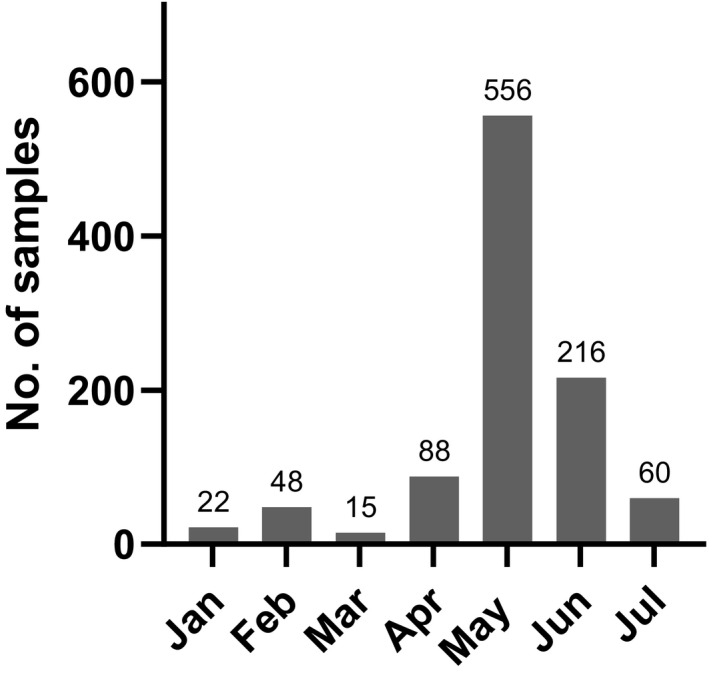
Sample distribution over the months (Germany and other European countries)

Between January and July 2020, cat sera (*n* = 1,005) were collected in an accredited laboratory in Germany as well as in a veterinary hospital in Berlin. The samples originated from Germany (91.7%) and other European countries (8.3%). Specimens were collected from all federal states in Germany (Table 1). Sera from an additional 21 European countries were collected between April and July 2020 (Table 2). The number of specimens taken per month varied between 22 and 556 sera (Figure 1). All samples were divided in three groups: Group 1 (*n* = 879) consisted of randomly chosen samples. Group 2 (*n* = 103) included specimens known to be highly seropositive for FCoV, these samples were gathered in Germany and other European countries. For Group 3 (*n* = 23), sera of cats from 17 different households were collected between 3rd of June and 16th of July 2020. Owners in the respective group either tested positive for SARS‐CoV‐2 by RT‐PCR (Gene E; *n* = 1), for specific antibodies by indirect ELISA (*n* = 12) or revealed positive results in both assays (*n* = 4) between the end of April and the end of May 2020. As left‐over sera from a diagnostic laboratory were used, the clinical status of the sampled cats remains unknown, with the exception of Group 3. In this cohort, symptoms of cats and owners have been documented.

**TABLE 1:** Number of samples taken per month and federal state within Germany

Federal state	January	February	March	April	May	June	July	In total
Baden‐Wuerttemberg	0	0	0	0	25	19 (1)	4	48 (1)
Bavaria	0	0	0	0	56 (1)	23 (1)	8 (2)	87 (4)
Berlin	13	43 (1)	10	72 (3)	87	33 (1)	2	260 (5)
Brandenburg	9	4	5	12 (1)	27 (1)	12	1	70 (2)
Bremen	0	0	0	0	1	0	0	1
Hamburg	0	0	0	0	3	0	0	3
Hesse	0	0	0	0	39 (1)	18	5	62 (1)
Lower Saxony	0	0	0	0	54 (1)	17 (1)	8	79 (2)
Mecklenburg‐Western Pomerania	0	0	0	0	9	3	0	12
North Rhine‐Westphalia	0	0	0	0	103	62 (1)	14 (1)	179 (2)
Rhineland‐Palatinate	0	0	0	2	39	16	5 (1)	62 (1)
Saarland	0	0	0	0	7	1	1	9
Saxony	0	0	0	0	4	1	0	5
Saxony‐Anhalt	0	1	0	0	8 (1)	2	0	11 (1)
Schleswig‐Holstein	0	0	0	0	18 (1)	3	2	23 (1)
Thuringia	0	0	0	0	4	6	1	11
In total	22	48	15	86	484	216	51	922

Note

The number of positive/inconclusive results confirmed by iIFT/sVNT is given in parenthesis

**TABLE 2:** Specimens collected per month in other European countries

Country	April	May	July	In total
Austria	2	7	0	9
Bulgaria	0	1	1	2
Croatia	0	1	0	1
Czech Rep.	0	7 (1)	1	8 (1)
Denmark	0	4	0	4
Estonia	0	3	2 (2)	5 (2)
Finland	0	2	0	2
France	0	3	0	3
Greece	0	2	1	3
Ireland	0	1	0	1
Latvia	0	0	2	2
Luxembourg	0	2	1	3
Netherlands	0	6	0	6
Norway	0	3	0	3
Poland	0	4	0	4
Romania	0	2	0	2
Slovakia	0	1	0	1
Slovenia	0	2	0	2
Spain	0	1	0	1
Sweden	0	7	1	8
Switzerland	0	13	0	13
In total	2	72	9	83

Note

Positive and inconclusive samples that were confirmed by iIFT/sVNT are shown in parenthesis

### 2.2 Nucleocapsid‐based ELISA (E_NC_)

A commercially available, double antigen, multispecies ELISA based on the nucleocapsid antigen provided by Innovative Diagnostics (kit: ID Screen SARS‐CoV‐2 Double Antigen Multispecies ELISA) was used according to the manufacturer's instructions. This assay was conducted a second time for samples that tested positive or inconclusive in either of the two ELISAs. Briefly, sera were added to microwells coated with the purified recombinant N protein. After an incubation period of 1 hr, plates were washed thrice. A purified N protein recombinant antigen horseradish peroxidase (HRP) conjugate was applied to bind to the free Fab of the anti‐SARS‐CoV‐2 antibodies bound to the coated N protein. Followed by another washing step, the substrate, 3,3′,5,5′‐tetramethylbenzidine (TMB) was added. The reaction was stopped by applying a stop solution (0.5 M H_2_SO_4_) after 20 min. The optical density was determined at a wavelength of 450 nm.

The following cut‐off values given by the manufacturer were used: Results with values <50 S/P% (samples to positive control ratio in percent) were considered as negative. Findings between 50 and 60 S/P% were recorded as doubtful, while values >60 S/P% were registered as positive. No information is available yet from the manufacturer or the literature with respect to sensitivity and specificity of this assay. Positive and negative controls were included in every ELISA plate.

### 2.3 Receptor‐binding domain‐based ELISA (E_RBD_)

The second ELISA applied in this study represented an RBD‐based multispecies ELISA performed as described previously (Wernike et al., 2020). In brief, sera diluted 1/100 in Tris‐buffered saline with Tween 20 (TBST) were incubated on wells coated with RBD antigen and uncoated wells simultaneously. Following a washing step with TBST, a multispecies conjugate (SBVMILK; obtained from ID Screen® Schmallenberg virus Milk Indirect ELISA; IDvet) was added. Another washing step was conducted, and TMB was used as substrate solution. After 10 min, a stop solution was added.

An optical density (OD) of ≤0.2 was considered as negative, values between 0.2 and 0.3 as inconclusive, while results ≥0.3 were rated as positive. This assay shows a specificity of 100% and a sensitivity of 98.3% using samples that had been taken from various experimentally infected or vaccinated animal species. Known positive and negative controls were included in all plates. Additionally, a known negative serum and an anti‐SARS‐CoV‐2 antibody‐positive bovine serum (Ulrich et al., 2020) were integrated in duplicates into every plate.

### 2.4 Indirect immunofluorescence test (iIFT)

To confirm the results of both ELISAs, indirect immunofluorescence test (iIFT) was performed. This assay was conducted for samples (*n* = 32), which yielded either positive or inconclusive results in one of the two ELISAs. Due to a lack of material, one of the respective sera could not be confirmed by iIFT. A recently developed assay was used (Schlottau et al., 2020). Vero E6 cells were infected with the 2019_nCoV Muc‐IMB‐1 SARS‐CoV‐2 isolate at a multiplicity of infection (MOI) of 0.1. Twenty‐four hours after infection, cells were fixed (4% paraformaldehyde) and permeabilized with 0.5% Triton‐X‐100. Heat inactivated and serially diluted sera were added. The cells were incubated for 1 hr. A washing step was conducted afterwards and an anti‐cat‐IgG‐FITC (Sigma‐Aldrich) antibody diluted 1/600 was applied. This was followed by washing the cell monolayer one more time. After adding a fluorescence preservation buffer, the assay was evaluated by fluorescence microscopy. Values ≥1/8 were considered as positive.

### 2.5 Surrogate virus neutralization test (sVNT)

In order to test for neutralizing antibodies (NAbs), a surrogate neutralization assay based on the receptor‐binding domain of the SARS‐CoV‐2 spike protein was used (Kit: GenScript cPass™ SARS‐CoV‐2 Neutralization Antibody Detection Kit). Due to lack of sample material, this test could only be performed for 19 out of 33 samples that tested positive or inconclusive in at least one of the two ELISAs. The assay was conducted according to manufacturer's instructions. Firstly, sera were diluted with sample dilution buffer (1:9). Subsequently, diluted samples were mixed with HRP‐RBD solution (1:1) and incubated for 30 min at 37°C. Per serum, 100 μl were pipetted to a well coated with human Angiotensin‐converting enzyme 2 (hACE2) protein. The plates were sealed and incubated for another 15 min at 37°C, followed by a washing step with washing solution for four times and application of 100 μl TMB solution. To stop the reaction, 50 μl of stop solution were added after 15 min and absorbances were measured at 450 nm. An inhibition rate <30% was considered negative, whereas values ≥30% were counted as positive. The conducted assay shows a sensitivity of 98.9% sensitivity and 98.8% specificity compared with plaque reduction neutralization test (PRNT) in a multispecies setting (Perera et al., 2020).

### 2.6 FCoV‐ELISA

The indirect ELISA, used to detect anti‐FCoV antibodies, is based on a native antigen obtained from Crandell‐Rees Feline Kidney Cells (Kit: VetLine Feline Corona Virus [FCoV/FIP] ELISA; FIPVT0870 [NovaTec Immundiagnostica GmbH]). This test was conducted according to the manufacturer's instructions. Samples of Group 2 (*n* = 55) were incubated on the coated plates. After washing, horseradish peroxidase (HRP) labelled Protein A/G conjugate was applied. Following another washing step, the substrate solution TMB was added. The reaction was stopped by using sulphuric acid (0.2 M) after 15 min. To evaluate the results, absorbance was investigated at 450 nm. Interpretation of findings was conducted in NovaTec Units (NTU).

Results <9 NTU were rated as negative, findings between 9 and 11 NTU were considered as doubtful and values >11 NTU were counted as positive. Findings were considered as highly positive if they reached values >30 NTU. According to the manufacturer, this test provides a sensitivity of >98% and a specificity of 92.31%.

**FIGURE 2:**
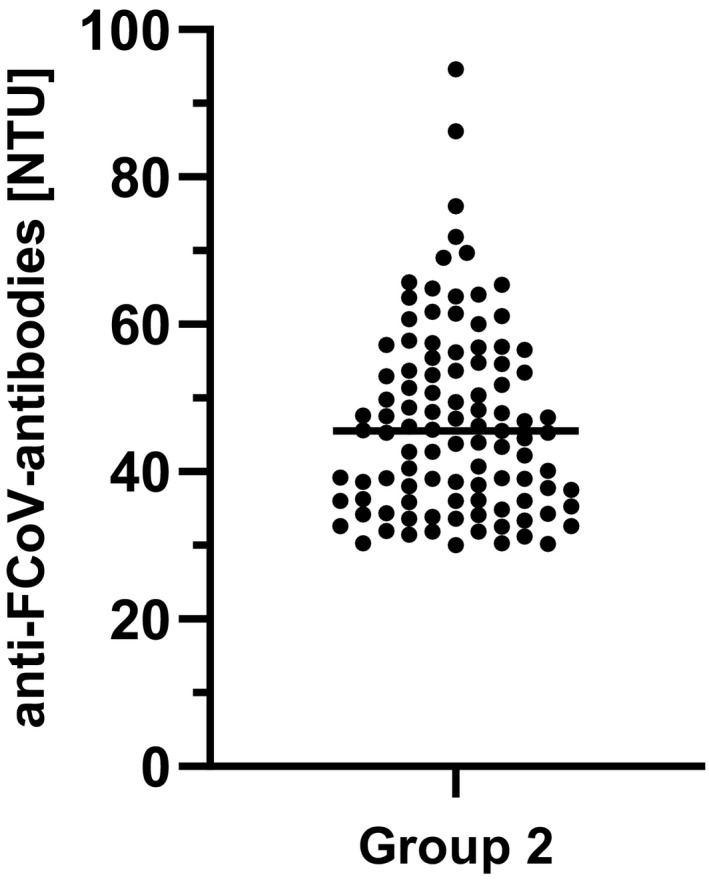
Anti‐FCoV antibodies in NovaTec Units (NTU) detected in Group 2 (*n* = 55)

All specimens (*n* = 103) included in Group 2 tested highly positive for FCoV antibodies with results between 30.01 and 94.64 NTU (Figure 2). Since sera originally belonging to Group 1 were re‐examined regarding the FCoV seropositivity to increase the number of highly FCoV‐positive samples, additional 78 samples were found to be positive for FCoV antibodies. Those sera did not reach the cut‐off value (>30 NTU) for high seropositivity and were therefore assigned to Group 1.

### 2.7 Statistical evaluation

GraphPad Prism 8 software, Microsoft Excel 2019 and IBM SPSS Statistics (Version 26) were used for data evaluation and visualization. Fisher's exact test was used to compare the different prevalence found in respective groups. For latter evaluation sera, which tested either positive in E_RBD_ and iIFT or inconclusive in E_RBD_ and positive in iIFT were considered as positive. Significance was defined as a *p*‐value < .05. All values are rounded to one decimal place.

## 3 RESULTS

### 3.1 Nucleocapsid ELISA (E_NC_)

**FIGURE 3:**
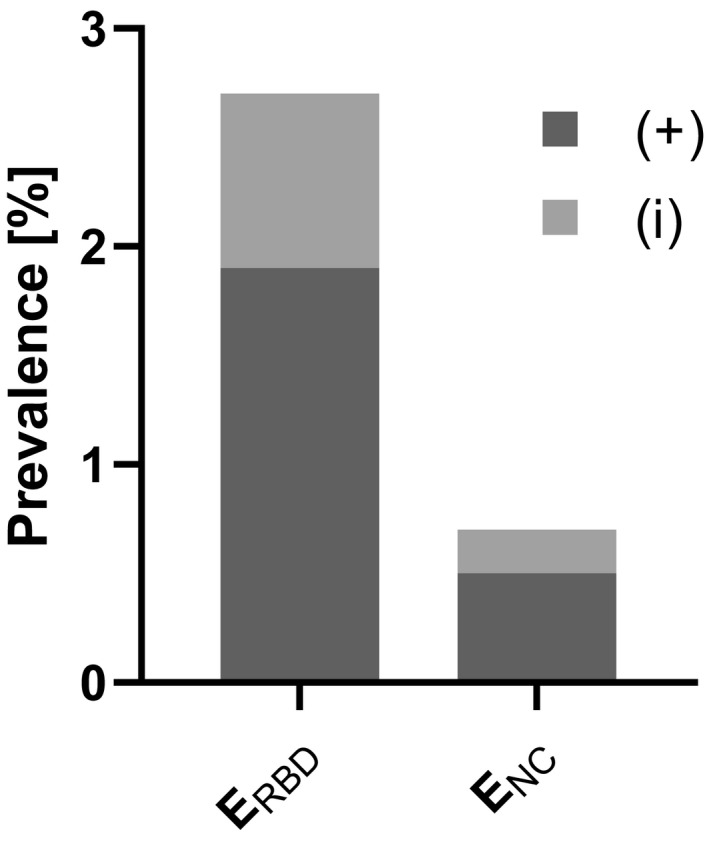
Overall prevalence considering positive (+) and inconclusive (i) samples detected by E_RBD_ (receptor‐binding domain‐based ELISA) and E_NC_ (nucleocapsid‐based ELISA) in Germany and other European countries

**FIGURE 4:**
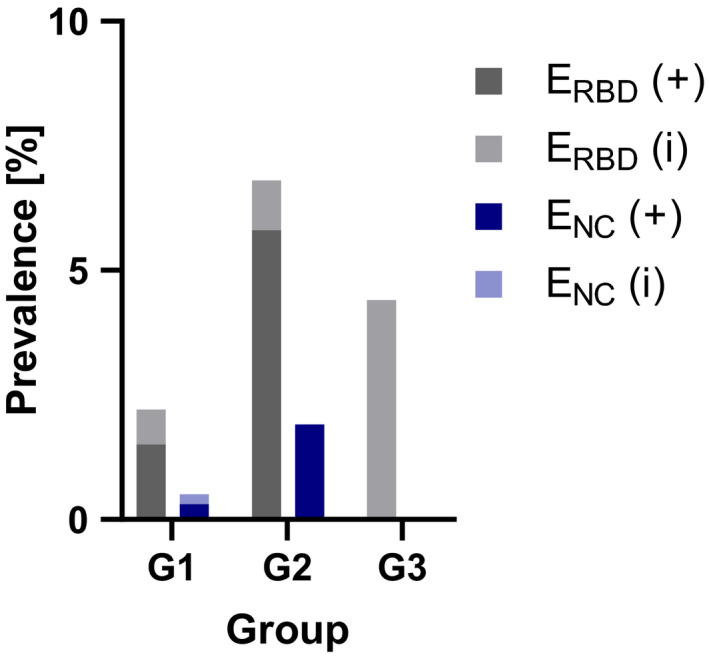
Positive (+) and inconclusive (i) specimens shown in percentage per group and assay. G1 (Group 1 = Randomly chosen samples), G2 (Group 2 = Anti‐FCoV antibody‐positive cats) and G3 (Group 3 = Cats with SARS‐CoV‐2‐positive owners)

**FIGURE 5:**
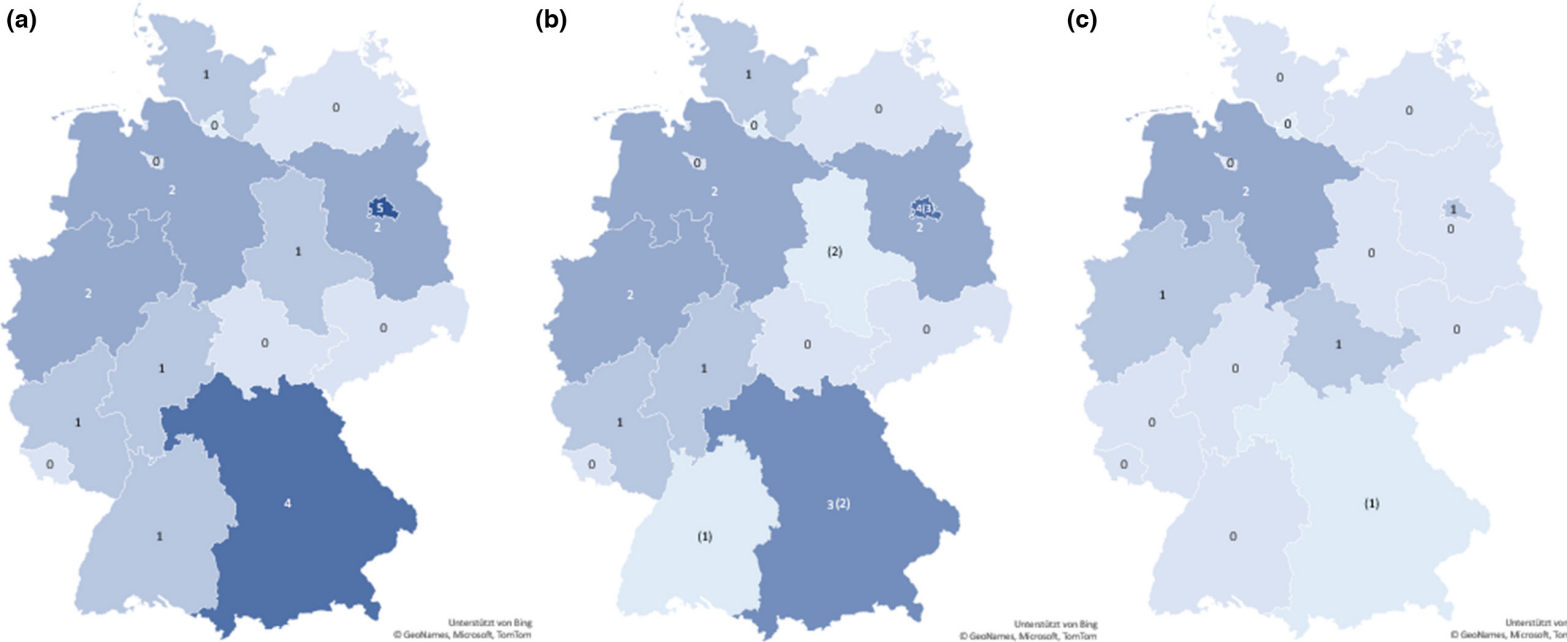
Origins of positive or inconclusive samples for anti‐SARS‐CoV‐2 antibodies within Germany. Doubtful results are displayed in parenthesis. (a) ELISA‐positive specimens confirmed by indirect immunofluorescence test (*n* = 20). (b) Sera which were found to be positive or inconclusive by E_RBD_ (*n* = 24). (c) Samples that tested positive or inconclusive in E_NC_ (*n* = 6)

The nucleocapsid‐based assay revealed five positive samples (0.5%; 95% CI: 0.1–1.0), while two specimens (0.2%; 95% CI: 0.0–0.5) yielded inconclusive results (Figure 3). In the randomly chosen specimens (Group 1), the E_NC_ test showed three (0.3%; 95% CI: 0.0–0.8) positive and two (0.2%; 95% CI: 0.0–0.6) inconclusive results. Whereas in the second group (FCoV antibody‐positive felids), two serum samples (1.9%; 95% CI: 0.0–4.9) tested positive for SARS‐CoV‐2 antibodies, no positive specimens were identified in Group 3 (cats with SARS‐CoV‐2 positive owners; Figure 4, Table S1). Antibody‐positive cats were detected in the German federal states Berlin (1/260), Lower Saxony (2/79), North Rhine‐Westphalia (1/179) and Thuringia (1/11). Inconclusive results were found for samples from Bavaria (1/87) in Germany (Figure 5) and Slovenia (1/2).

### 3.2 RBD ELISA (E_RBD_)

The E_RBD_ revealed a seroprevalence of 1.9% (19/1,005; 95% CI: 1.1–2.8) in the tested samples and 8 (0.8%; 95% CI: 0.3–1.4) inconclusive results (Figure 3). In the randomly chosen specimens (Group 1), 13 (1.5%; 95% CI: 0.8–2.4) positive and 6 (0.7%; 95% CI: 0.2–1.3) inconclusive samples were identified. Prevalence in Groups 2 and 3 were higher with six (5.8%; 95% CI: 1.9–10.7) positive and one (1%; 95% CI: 0.0–3.9) inconclusive finding in Group 2, and no positive but one (4.4%; 95% CI: 0.0–13.0) inconclusive sample in Group 3 (Figure 4, Table S1). Distribution of positive cases was clearly different from that identified by the E_NC_. The RBD‐based ELISA revealed positive cases in the German federal states Bavaria (3/87), Berlin (4/260), Brandenburg (2/70), Hesse (1/62), Lower Saxony (2/79), North Rhine‐Westphalia (2/179), Rhineland‐Palatinate (1/62) and Schleswig‐Holstein (1/23). Furthermore, inconclusive specimens were observed in Baden‐Wuerttemberg (1/48), Bavaria (2/87), Berlin (3/260) and Saxony‐Anhalt (2/11; Figure 5). Positive specimens were also detected in Estonia (2/5) and the Czech Republic (1/8).

### 3.3 Indirect immunofluorescence test (IIFT)

Sera (32/1,005) which yielded positive or inconclusive results in either of the two ELISAs used in this study, were tested by iIFT in order to confirm the results. Respective samples revealed 23 positive specimens. Differentiated in groups, Group 1 (randomly chosen sera) yielded 16 seropositive samples, while 6 specimens tested positive in Group 2 (FCoV‐ antibody‐positive cats). In the last group, one serum showed a positive result. All positive specimens revealed values between 1/8 and >1/32. The distribution of positive cases was similar to that obtained with the E_RBD_. Antibody‐positive cats were found in Baden‐Wuerttemberg (1/48), Bavaria (4/87), Berlin (5/260), Brandenburg (2/70), Hesse (1/62), Lower Saxony (2/79), North Rhine‐Westphalia (2/179), Rhineland‐Palatinate (1/62), Saxony‐Anhalt (1/11) and Schleswig‐Holstein (1/23; Figure 5). Additionally, all sera from European countries identified as positive by E_RBD_ were confirmed by iIFT.

**FIGURE 6:**
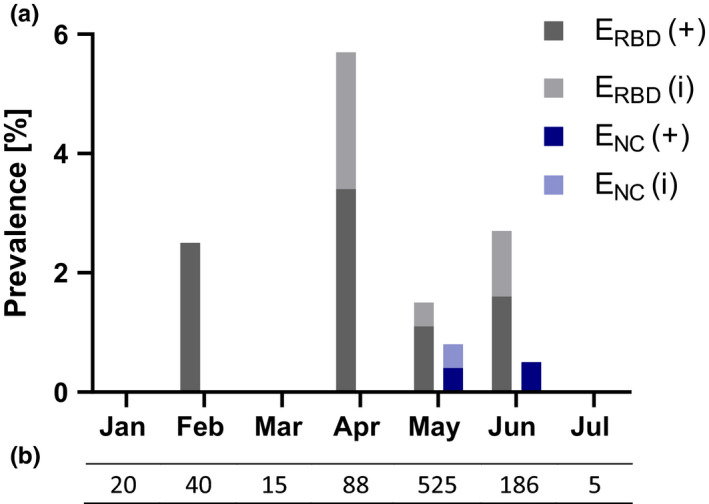
(a) Prevalence of positive (+) and inconclusive (i) samples per month and assay detected in Group 1 (Randomly chosen specimens) considering Germany and other European countries. (b) Number of sera collected per month

In January and March, no positive or inconclusive results were found either in using the ELISAs or by iIFT. Considering Group 1, specimens screened in February and April revealed positive and inconclusive results only when tested by E_RBD_ and iIFT. In February, 1 positive sample (2.5%) was found in the respective tests, whereas in April, 3 (3.4%) specimens tested positive in E_RBD_. Moreover, 2 sera (2.3%) yielded inconclusive values in the E_RBD_. By iIFT, four sera tested positive in the respective month. The number of positive samples in May varied between 2 (0.4%) in E_NC_ and 6 (1.1%) in E_RBD_, while the results were inconclusive in 0.4% of the cases (*n* = 2) in both ELISAs. Of the eight positive or inconclusive samples detected by RBD‐based ELISA in May, six were confirmed as positive by iIFT. In June, the prevalence increased slightly, one specimen tested positive (0.5%) in E_NC_, three (1.6%) in E_RBD_. Samples with inconclusive readings were identified using E_RBD_ in 2 cases (1.1%). In this month, four of the samples that tested positive or inconclusive in E_RBD_ revealed positive results by iIFT. In July, no positive or doubtful sera were detected in Group 1 (Figure 6, Table S1).

The agreement of both ELISAs regarding positive samples was 4.4%. Only one specimen tested positive in both respective tests and the iIFT. The latter confirmed 100% of positive results and showed anti‐SARS‐CoV‐2 positivity for 57.1% (4/7) of inconclusive findings of the RBD‐based ELISA (E_RBD_), while E_NC_/iIFT revealed an agreement of 3.7% in positive findings and no inconclusive result could be confirmed (Table 3, Table S1).

**TABLE 3:** Agreement of different assays in absolute numbers and %

Agreement (%)										
Results	E_NC_/E_RBD_		E_NC_/iIFT		E_RBD_/iIFT		E_NC_/sVNT		E_RBD_/sVNT	
	No.	%	No.	%	No.	%	No.	%	No.	%
Negative	972 (998/978)	96.8	3 (25/9)	9.7	6 (6/9)	66.7	2 (13/8)	10.5	6 (6/8)	75.0
Positive	1 (5/19)	4.4	1 (5/23)	3.7	19 (19/19)	100.0	0 (4/11)	0.0	9 (9/9)	100.0
Inconclusive	0 (2/8)	0.0	0 (2/0)	0.0	4 (7/4)^a^	57.1	0 (2/0)	0.0	2 (4/2)	50.0
Samples tested	1,005		32		32		19		19	

Note

The number of samples that tested positive or inconclusive in the respective tests are shown in parenthesis. Only one serum that yielded positive results in E_NC_ was confirmed by E_RBD_ and iIFT.

^a^ Due to lack of material, one sample that resulted inconclusive in E_RBD_ could not be confirmed by iIFT and is therefore excluded from the calculation.

### 3.4 Surrogate virus neutralization test (SVNT)

Samples that revealed positive or inconclusive results in one of the previously conducted tests were screened for neutralizing antibodies. The material of 14/33 sera was not sufficient to perform sVNT. This assay confirmed the positive results of iIFT and E_RBD_ (9/9) with an agreement of 100%, while 50% of samples that tested inconclusive (2/4) in E_RBD_ were approved. Furthermore, no positive or inconclusive results yielded in E_NC_ could be confirmed (Table 3, Table S1).

Our results indicated substantial deficits in the sensitivity and specificity of the nucleocapsid‐based commercial assay in our analysis of feline sera. Furthermore, the higher prevalence of SARS‐CoV‐2 antibody‐positive cats in the anti‐FCoV antibody‐positive group could be connected to a cross‐reactivity of FCoV‐ELISA for SARS‐CoV‐2 antibodies.

### 3.5 Statistical evaluation

Fisher's exact test showed a significant difference (*p* = .0212) in prevalence between Group 1 (randomly chosen samples) and Group 2 (FCoV antibody‐positive cats). While no statistical significance (*p* = .358) was detected comparing the prevalence of Group 1 and Group 3 (cats with SARS‐CoV‐2‐positive owners).

## 4 DISCUSSION

Published data suggest that among companion animals, cats exhibit a moderate to high susceptibility to SARS‐CoV‐2 infection. Consistent with this assessment, prolonged viral shedding and higher seroconversion rates have been reported in felids when compared with dogs (Bosco‐Lauth et al., 2020). Therefore, domestic cats were focus of interest of this large‐scale survey.

Various factors seem to have an impact on SARS‐CoV‐2 antibody prevalence in cats. It was shown in previous studies that the number of seroconverted felids correlated with the prevalence of SARS‐CoV‐2 infections in humans (Michelitsch et al., 2020; Patterson et al., 2020). Two investigations, one performed during a phase of low prevalence in humans, and another conducted during the second wave in Germany, revealed an overall seroprevalence of 0.69% and 1.36%, respectively using an RBD‐based ELISA (Michelitsch et al., 2020; Michelitsch, Schön, et al., 2021). Additionally, a survey screening the percentage of SARS‐CoV‐2 antibody‐positive cats in Italy mainly focused on highly affected regions such as Lombardy exhibited a higher prevalence of 5.8% (Patterson et al., 2020). Surveys conducted in the same regions with different sample cohorts suggest that different husbandry and living conditions of cats have an additional impact on seroprevalence (Deng et al., 2020; Zhang, Zhang, et al., 2020).

This early‐phase study in the COVID‐19 pandemic with samples taken between January and July 2020 showed an overall prevalence of 1.9% (95% CI: 1.1–2.8) positive and 0.8% (95% CI: 0.3–1.4) inconclusive results in E_RBD_. Similar to previously published investigations, this study also suggests a connection between case numbers in humans and antibody prevalence in domestic cats. In Group 1, 2.5% of positive cats were found in February 2020 using E_RBD_. Prevalence increased in April to 3.4% positive and 2.3% inconclusive samples as assessed by E_RBD_, while they were decreasing in May and June with values between 1.1% and 1.6%. As only a small number of samples was screened in January (*n* = 22) and March (*n* = 15), it is not surprising that no positive cases were observed in these months. The varying amount of sera screened per group and month must be considered as a limitation of this study. In felids, specific antibody responses against SARS‐CoV‐2 are detectable already around day 10 after infection (Shi et al., 2020). The ELISA (E_RBD_) used gave highly reliable results from day 8 after experimental or natural infection of various species onwards, with a sensitivity of 98.3% and a specificity of 100% (Wernike et al., 2020). Considering these points, the seropositivity peaking in April indicates a correlation with the prevalence of SARS‐CoV‐2 infection in humans, as the first wave in Germany reached its peak around the16th of March 2020 (Robert Koch‐Institute, 2020). By the end of July 2020, 208.698 cases of SARS‐CoV‐2 infections in humans were detected in Germany and reported by the Robert Koch‐Institute, which means only 0.25% of the population had been registered as infected to that date. It must be taken into account that reported and confirmed cases are dependent on the number of tests conducted. Thus, the estimated number of infections is higher. Model calculations suggested, for example, that 0.85% of the population in Germany were infected by the 4th of May 2020 (Flaxman et al., 2020). Seroconversion studies in humans including all regions of Germany have not been conducted during the first wave. However, two investigations performed between March and May 2020 (Fischer et al., 2020) and between July and December 2020 (Gornyk et al., 2021) revealed a seroconversion rate between 1% and 2%. Hence, both studies found similar results to the one we found in cats, which highlights again, that cats are a valuable indicator concerning the prevalence of SARS‐CoV‐2 antibodies in the human population.

The decreasing SARS‐CoV‐2 seroprevalence observed in cats in May and June, might suggest a short‐ rather than long‐lived immunity. First hints for short‐duration antibody responses in felids were reported by Schulz et al. (2021) and Zhang, Zhang, et al. (2020). The specific antibodies targeted against RBD peaked around the 5th and 2nd week of surveillance and were detectable up to the 10th and 15th week of surveillance, respectively. Neutralizing antibodies revealed a similar trend. So far, the development of antibody titres was only investigated in a small number of cats which were naturally infected. Our survey does not provide data for titre development in individuals as testing was performed without any knowledge about time of antigen contact and only one sample was investigated per animal. Further studies are needed to determine whether and to what extent a specific long‐term antibody response in felids may be present.

The group of cats (*n* = 23) with owners that tested positive for SARS‐CoV‐2 by RT‐PCR and/or ELISA revealed a single inconclusive result using E_RBD,_ which was confirmed as positive by iIFT. No respiratory or gastrointestinal clinical signs had been observed in this cat. Further information towards the clinical status of cats included in Group 3 can be found in Table S2. While the prevalence of 4.4% (95% CI: 0.0–13.0) is higher than the average prevalence reported in Group 1, the difference is not significant (*p* = .358). Therefore, our findings are consistent with those reported by Temmam et al. (2020) and Patterson et al. (2020). In contrast, a study performed by Fritz et al. (2021) showed a significantly higher prevalence of anti‐SARS‐CoV‐2 antibodies in cats kept in COVID‐19 households. The time interval between the suspected infection and the antibody test seems to be an important factor for the seroreactivity found in cats (Schulz et al., 2021; Zhang, Zhang, et al., 2020). In cases where animal owners were tested positive for viral RNA (*n* = 5), we conducted the antibody tests in their cats around 6 weeks after the positive RT‐PCR test result of the owner. A limitation of our study is that 12 of the owners only tested positive for anti‐SARS‐CoV‐2 antibodies. Hence, time interval between a possible infection and the sampling of the cats remains unknown. Furthermore, the varying seroprevalence found in cats with positive owners could be caused by diverging living conditions of the screened felids and different tests and cut‐off values used in these studies.

Endemic coronaviruses such as FCoV are widespread in cats (Pedersen, 1976). Thus, cross‐reactivity must be taken in consideration when evaluating the specificity of serological tests. We included sera that were highly positive for FCoV antibodies to define existence and extent of cross‐reactivity. It is worth noting that our results show a significant difference (*p* = .0212) in prevalence between randomly chosen samples (1.8%; 95% CI: 1.0–2.7) and specimens known to contain high antibody levels to FCoV (5.8%; 95% CI: 1.9–10.7). Cross‐reactivity of the RBD‐based ELISA for FCoV antibodies was excluded during development and test validation (Michelitsch et al., 2020; Wernike et al., 2020). Hence, our results might indicate cross‐reactivity of SARS‐CoV‐2 antibodies in the FCoV‐ELISA used here. It remains unknown whether the cats screened in Group 2 suffered from acute feline infectious peritonitis or only got in contact with the pathogen and therefore mounted an antibody response. However, co‐infections with certain pathogens are known to be predisposing factors for increased morbidity and mortality in humans (Touzard‐Romo et al., 2020; Wu et al., 2020; Zhu, Cao, et al., 2020). Thus, one hypothesis to explain the differences found here, might be a higher susceptibility for SARS‐CoV‐2 infection of FCoV‐positive cats. A study examining the prevalence of SARS‐CoV‐2 and co‐infections reported no significant correlation between seropositivity for SARS‐CoV‐2 and infections with feline immunodeficiency virus (FIV), feline leukaemia virus (FeLV), *Toxoplasma gondii* or *Leishmania infantum* (Villanueva‐Saz et al., 2021). While prolonged virus shedding was observed in a cat that additionally tested positive for FIV (Schulz et al., 2021) and one cat with chronic rhinosinusitis (Neira et al., 2021). Nevertheless, both studies were limited by the small number of samples. So far, there are no studies indicating that FCoV infections are associated with a higher susceptibility for SARS‐CoV‐2. Furthermore, prevalence studies screening for FCoV in order to exclude cross‐reactivity of the tests used did not find any hints for a higher prevalence in FCoV‐positive cats (Michelitsch et al., 2020; Villanueva‐Saz et al., 2021).

Whereas sVNT and iIFT confirmed 100% of positive and 50%–57.1% of inconclusive samples detected by E_RBD_, the agreement was significantly lower between sVNT/iIFT and E_NC_. The nucleocapsid‐based assay (E_NC_) showed an agreement of 0%–3.7% for positive specimens and 0% for inconclusive specimens regarding sVNT and iIFT. Due to the clear differences found and in order to test for reproducibility of the results, all samples were analysed in a repeat experiment using the nucleocapsid‐based assay. The chosen cut‐off value could be excluded as reason for lack of sensitivity, as results of samples that tested positive in E_RBD_ or iIFT revealed values between <0.1 and 30.9 S/P% in E_NC_. Assays detecting antibodies targeting nucleocapsid antigen are known to show higher cross‐reactivity in humans, whereas RBD‐based assays feature higher specificity (Amanat et al., 2020; Chia et al., 2020). Additionally, a meta‐analysis performed by Kontou et al. (2020) revealed lower sensitivity and specificity of nucleocapsid‐based assays when compared with RBD‐based ELISAs. These results were confirmed for endemic coronaviruses in animals as well (Kim et al., 2020). However, contradicting evidence was reported by Dileepan et al. (2021). In that study, a different nucleocapsid‐based assay showed higher specificity and sensitivity than an RBD‐based ELISA. Nevertheless, sensitivity and specificity are not only depending on the target but differ between the specific tests (Rikhtegaran Tehrani et al., 2020). For instance, the type of nucleocapsid protein can have an impact on the assay sensitivity. Di et al. (2021) developed ELISAs with two different recombinant forms of the nucleocapsid protein and found that the RNA‐bound type showed a lower sensitivity than the one free of RNA. As a commercially available test was used here, quality of the nucleocapsid antigen used remains unknown. Regardless of the nucleocapsid protein quality nucleocapsid antigen could be susceptible to degradation, if handled inappropriately. This instability could lead to decreased sensitivity and specificity. As the assay was used exactly according to manufacturer's instructions, handling mistakes can be excluded.

Furthermore, the commercially available nucleocapsid‐based assay used here was included in other studies before. Two of them, conducted in Italy, revealed a prevalence of 1% (1/105) and 0% (0/99) (Spada et al., 2021; Stranieri et al., 2021). Another one, performed in Thailand, found a prevalence of 0.36% (4/1,112) (Udom et al., 2021). Moreover, an investigation of 17 cats with owners that previously tested positive for SARS‐CoV‐2 was performed (Neira et al., 2021). While positive antibody findings were confirmed by previous positive RT‐PCR results in the latter study (Neira et al., 2021), no additional tests were used to confirm the results in the study performed by Spada et al. (2021). Additionally, a VNT conducted by Udom et al. (2021) did not confirm any of the 4 seropositive samples found by this nucleocapsid‐based assay. This coincides with the results of our study in which the same nucleocapsid‐based ELISA was tested against an RBD‐based ELISA as well as an iIFT and a sVNT. The latter are known to show high sensitivity and specificity. Only 1 sample that tested positive by the nucleocapsid‐based assay was confirmed by iIFT and E_RBD_, while several false‐positive and false‐negative results were detected. Thus, this nucleocapsid‐based ELISA reveals neither sufficient sensitivity nor specificity for detection of anti‐SARS‐CoV‐2 antibodies in cats.

Despite a similar seroconversion rate in cats compared with humans during the first wave of infection, we detected only a small number of positive animals even after direct contact to a SARS‐CoV‐2 infected person. Moreover, no cat to human transmissions were observed so far. This suggests that cats are a negligible entity with respect to virus transmission. It may nevertheless be advisable for SARS‐CoV‐2‐positive cat owners to ensure sufficient distance and hygiene to avoid infection of their cats (Hosie et al., 2021).

## CONFLICT OF INTEREST

The authors declare no conflict of interest.

## ETHICAL APPROVAL

The cat sera used in this study were left‐over sera collected in a Laboratory for veterinary diagnostics and a small animal clinic in Germany. Therefore, the study design does not meet the German criteria for an animal experiment. Hence ethical approval is not required for this study.

## Supporting information

Table S1‐S2

## Data Availability

The data that support the findings of this study are available from the corresponding author upon reasonable request.
