# Supplementary material for: Prevalence of anti‐severe acute respiratory syndrome coronavirus 2 antibodies in cats in Germany and other European countries in the early phase of the coronavirus disease‐19 pandemic
Source: Zoonoses Public Health. 2022 Mar 2;69(5):439–50. doi: 10.1111/zph.12932 (PMC9115359; doi:10.1111/zph.12932)
Supplement: Supplementary file 1 — Table S1‐S2 [file ZPH-69-439-s001.docx]

**Prevalence of anti‐severe acute respiratory syndrome coronavirus 2 antibodies in cats in Germany and other European countries in the early phase of the coronavirus disease‐19 pandemic**

*Julia Maria Adler^1,2,^*^6^*, Corinna Weber^2^, Kerstin Wernike^3^, Anna Michelitsch^3^, Karin Friedrich^2^, Jakob Trimpert^1^, Martin Beer^3^, Barbara Kohn^5^, Klaus Osterrieder^1,4,^*^*^*, Elisabeth Müller^2,^*^*^

*^1^ Institut für Virologie, Freie Universität Berlin, 14163 Berlin, Germany ^2^ Laboklin GmbH & Co.KG, 97688 Bad Kissingen, Germany ^3^ Institute of Diagnostic Virology, Friedrich-Loeffler-Institut, 17493 Greifswald–Insel Riems, Germany ^4^ Department of Infectious Diseases and Public Health, Jockey Club College of Veterinary Medicine and Life Sciences, City University of Hong Kong, Kowloon, Hong Kong ^5^ Klinik für kleine Haustiere, Freie Universität Berlin, 14163 Berlin, Germany*

^6^ *Present Address:* *Department of Infectious Diseases and Respiratory Medicine, Charité, Universitätsmedizin Berlin, 10117 Berlin, Germany*

^*^ *Authors to whom correspondence should be addressed.*

**Correspondence**

Klaus Osterrieder, Institut für Virologie, Freie Universität Berlin, 14163 Berlin, Germany.

Email: no.34@fu-berlin.com

Elisabeth Müller, Laboklin GmbH & Co.KG, 97688 Bad Kissingen, Germany.

Email: mueller@laboklin.com

**Supplementary Material**

**Table S1.** Overview of test results. Only samples that yielded positive (+) or inconclusive (i) results in at least one of the conducted assays are listed. (n.t. = not tested due to lack of material)

| **Sample** | | | **Results** | | | |
| --- | --- | --- | --- | --- | --- | --- |
| **No.** | **Group No.** | **Sampling date** | **E_NC_** | **E_RBD_** | **iIFT** | **sVNT** |
| 1 | 1 | 02.2020 | - | + | + | + |
| 2 | 1 | 04.2020 | - | + | + | n.t. |
| 3 | 1 | 04.2020 | - | + | + | + |
| 4 | 1 | 04.2020 | - | (i) | + | n.t |
| 5 | 1 | 04.2020 | - | + | + | n.t |
| 6 | 1 | 04.2020 | - | (i) | n.t. | n.t |
| 7 | 1 | 05.2020 | - | (i) | - | - |
| 8 | 1 | 05.2020 | - | (i) | - | n.t |
| 9 | 1 | 05.2020 | - | + | + | + |
| 10 | 1 | 05.2020 | - | + | + | + |
| 11 | 1 | 05.2020 | (i) | - | - | - |
| 12 | 1 | 05.2020 | + | - | - | - |
| 13 | 1 | 05.2020 | - | + | + | + |
| 14 | 1 | 05.2020 | + | - | - | - |
| 15 | 1 | 05.2020 | - | + | + | + |
| 16 | 1 | 05.2020 | - | + | + | + |
| 17 | 1 | 05.2020 | (i) | - | - | - |
| 18 | 1 | 05.2020 | - | + | + | + |
| 19 | 1 | 05.2020 | - | (i) | + | + |
| 20 | 1 | 06.2020 | + | - | - | - |
| 21 | 1 | 06.2020 | - | + | + | n.t |
| 22 | 1 | 06.2020 | + | - | - | - |
| 23 | 1 | 06.2020 | - | (i) | + | + |
| 24 | 1 | 06.2020 | - | (i) | - | - |
| 25 | 1 | 06.2020 | - | + | + | n.t |
| 26 | 1 | 06.2020 | - | + | + | n.t |
| 27 | 2 | 07.2020 | - | + | + | n.t |
| 28 | 2 | 07.2020 | - | + | + | n.t |
| 29 | 2 | 07.2020 | - | + | + | n.t |
| 30 | 2 | 07.2020 | - | + | + | n.t |
| 31 | 2 | 07.2020 | + | + | + | n.t |
| 32 | 2 | 07.2020 | - | + | + | + |
| 33 | 3 | 06.2020 | - | (i) | + | n.t. |

**Table S2.** Clinical status and test results of cats assigned to group 3 (cats with SARS-CoV-2 positive owners). All cats that showed respiratory symptoms tested negative (-) in all assays, whilst 1 cat without clinical signs yielded positive (+) results. Cat 6 to 23 were either asymptomatic or the clinical status was unknown.

| **No.** | **Symptoms** | **Results** | | |
| --- | --- | --- | --- | --- |
|  |  | **E_NC_**^†^ | **E_RBD_**^†^ | **iIFT**^†^ |
| Cat 1 | Cough | - | - | - |
| Cat 2 | 3 weeks before testing: sniffles and upper respiratory symptoms for about 1 week | - | - | - |
| Cat 3 | 4 weeks before testing: mild lower respiratory tract symptoms such as coughing | - | - | - |
| Cat 4 | in April: sniffles and upper respiratory symptoms for about 10 days | - | - | - |
| Cat 5 | no symptoms observed | - | + | + |
| Cat 6 - 23 | no symptoms observed | - | - | - |

^†^ E_NC_ = nucleocapsid-based ELISA, E_RBD_ = receptor-binding-domain-based ELISA, iIFT = indirect immunofluorescence test
